# Degradation strategy of cyclin D1 in cancer cells and the potential clinical application

**DOI:** 10.3389/fonc.2022.949688

**Published:** 2022-08-18

**Authors:** Shuyi Chen, Ling Li

**Affiliations:** ^1^ The Sixth Student Battalion, School of Basic Medical Sciences, Fourth Military Medical University, Xi’an, China; ^2^ Department of Cell Biology, National Translational Science Center for Molecular Medicine, Fourth Military Medical University, Xi’an, China

**Keywords:** cyclin D1 degradation, cancer cells, FBX4, combination mode, photodynamic therapy

## Abstract

Cyclin D1 has been reported to be upregulated in several solid and hematologic tumors, promoting cancer progression. Thus, decreasing cyclin D1 by degradation could be a promising target strategy for cancer therapy. This mini review summarizes the roles of cyclin D1 in tumorigenesis and progression and its degradation strategies. Besides, we proposed an exploration of the degradation of cyclin D1 by FBX4, an F box protein belonging to the E3 ligase SKP-CUL-F-box (SCF) complex, which mediates substrate ubiquitination, as well as a postulate about the concrete combination mode of FBX4 and cyclin D1. Furthermore, we proposed a possible photodynamic therapy strategythat is based on the above concrete combination mode for treating superficial cancer.

## Introduction

Loss of cell cycle control usually drives rapid and uncontrollable tumor growth, which has been considered an important feature for cancer cells (1). Cyclin D1, a key regulator of cell cycle progression, can integrate extracellular mitotic signals into DNA synthesis by binding to cyclin-dependent kinase 4/6 (CDK4/6), thus facilitating the cell cycle switch from G1 to S phase.

As its name suggests, cyclin D1 protein abundance fluctuates periodically throughout the cell cycle. In the late G1 phase, cyclin D1 levels increase and then activate CDK4/6, which helps trigger the progress of the cell cycle. In the S phase, however, the level of cyclin D1 falls. Cyclin D1 controls the G1-S phase transition in both CDK-dependent and CDK-independent manners.

**Figure 1:**
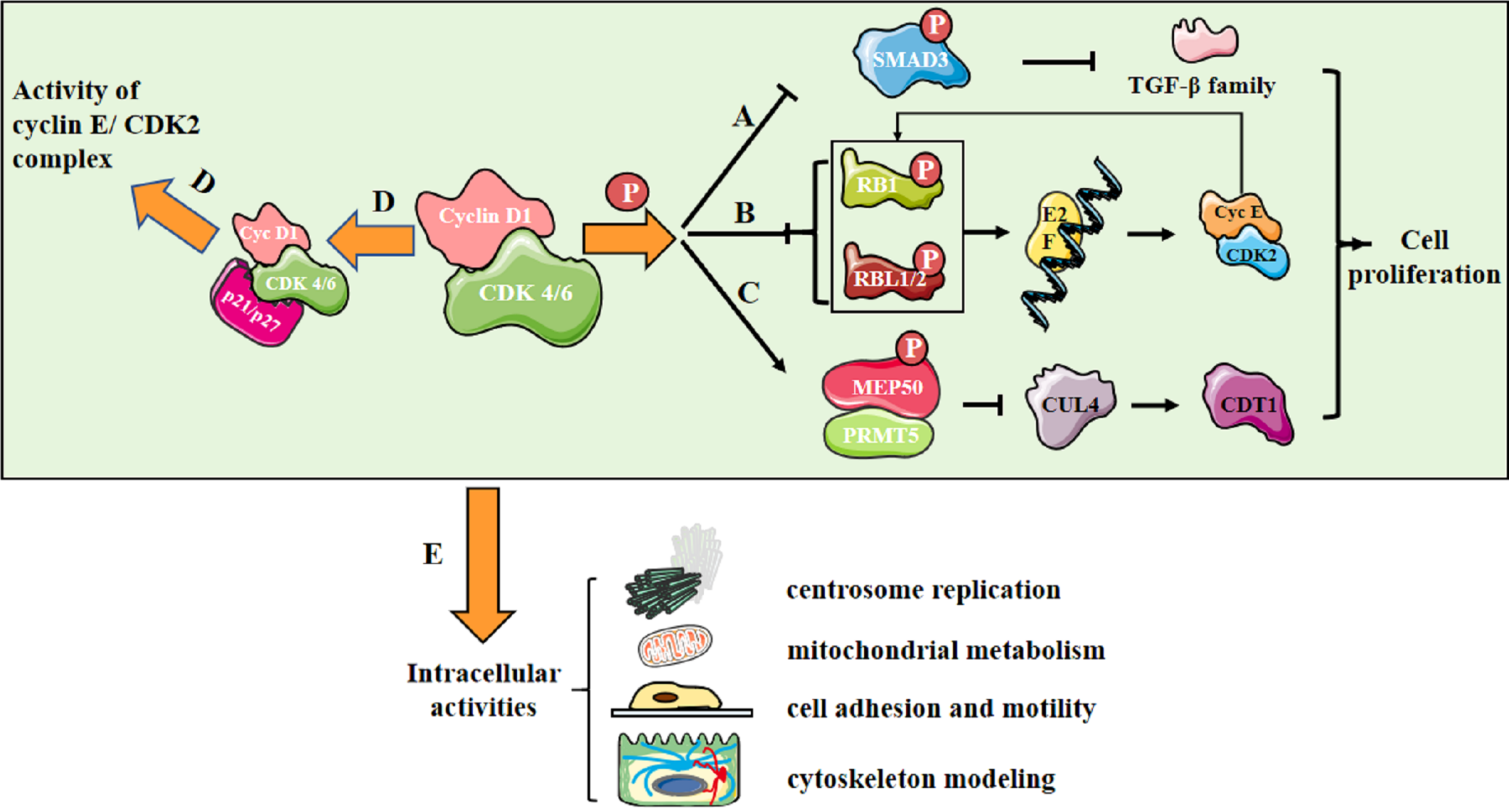
Cyclin D1/CDK4/6 promotes cell proliferation. **(A)** Cyclin D1/CDK 4/6 phosphorylates SMAD3 and downregulates the transcription of TGF-β family genes involving in growth inhibition; **(B)** Cyclin D1/CDK 4/6 phosphorylates RB1 and RBL1/2, leading to their inactivation and release of E2F family. E2F controls the transcription of genes essential for cyclin E synthesis, which will activate CDK2 and further promote RB phosphorylation, forming a positive feedback loop; **(C)** Cyclin D1/CDK4/6 triggers an increase in MEP50/PRMT5 activity and a decrease in the activity of CUL4, the E3 ligase of CDT1 and the replication licensing protein at the meantime; **(D)** Cyclin D1/CDK 4/6 sequesters the CDK inhibitors(CKIs) p21^CIP1^ and p27^KIP1^, indirectly upregulating cyclin E/CDK2 activity; **(E)** Molecular activities mediate intracellular activities, including centrosome replication, mitochondrial metabolism, cell adhesion and motility, and cytoskeleton modeling.

In CDK-dependent manner, the cyclin D1/CDK4/6 complex phosphorylates SMAD3 and downregulates TGF-β family (2) (**Figure 1A**); the complex also phosphorylates RB1 and RBL1/2 and promotes E2F release, then upregulates cyclin E/CDK2 levels, further resulting in RB phosphorylation (3) (**Figure 1B**). Besides, the complex phosphorylates MEP50/PRMT5 and upregulates CDT1 activity *via* downregulating CUL4 activity (4) (**Figure 1C**). Additionally, the cyclin D1/CDK4/6 complex sequesters p21 and p27, indirectly upregulating cyclin E/CDK2 activity (5) (**Figure 1D**). All these molecular events mediate intracellular activities, including centrosome replication, mitochondrial metabolism, cell adhesion and motility, and cytoskeleton modeling (3) (**Figure 1E**).

In addition to CDK-dependent cell cycle regulation activity, cyclin D1 itself has CDK-independent effects. Cyclin D1 is involved in the DNA damage response by interacting with RAD51 and BRCA2 (6). Besides, cyclin D1 regulates cell growth and differentiation by binding to several transcription factor families including nuclear hormone receptor family members [estrogen receptor-α (ERα), androgen receptor (AR), peroxisome proliferator-activated receptor-γ (PPAR γ)) and their co-activators (SRC1, GRIP1, and AIB1), BMYB, and the MYB-related transcription factor DMP1, the helix–loop–helix transcription factors neurogenic differentiation factor 1 (NEUROD1), as well as MYOD and C/EBPβ] (7–9). Cyclin D1 also promotes cell cycle progression by binding to signal transducer and activator of transcription 3 (STAT3) and TAFII250 (3).

Generally, all those activities are related to G1-S phase transition and cell proliferation. When dysregulated, upregulated cyclin D1 antagonizes the checkpoint induced cell cycle arrest, disturbs DNA replication, and allows damaged DNA to be replicated (4, 10, 11). Therefore, the overexpression of cyclin D1 or the failure of cyclin D1 degradation both accelerate G1-S transition, helping cancer cells to gain a survival advantage and an uncontrolled proliferation, which further promotes the invasiveness and malignance of cancer.

Cyclin D1 upregulation has been seen in at least 11 kinds of cancers, including head and neck squamous cell carcinoma (12), non-small-cell lung cancer (NSCLC) (13), endometrial cancer (14), melanoma (15), pancreatic cancer (16, 17), gastric cancer (18), breast cancer (19), colorectal cancer (20), mantle cell lymphoma (21), multiple myeloma (22), and prostate cancer (23). The prevailing view is that high levels of cyclin D1 expression have a positive correlation with poor prognosis in a wide variety of tumors such as nasopharyngeal carcinoma (NPC) (24), gastric cancer tissues (25), and squamous cell carcinoma of the head and neck (12, 26). Interestingly, a minority of reports suggest that a high level of cyclin D1 expression is associated with a favorable prognosis in breast cancer and clear renal cell carcinoma (27, 28).

Usually, a high level of cyclin D1 derives from enhanced mRNA expression of cyclin D1, splice variants, transcript aberrations, and downregulated cyclin D1 degradation (29–32), among which the dysregulated degradation of cyclin D1 could be a significant reason (3). In the early stage of cancer, failure to degrade cyclin D1 after double-strand DNA breaks (DSBs) may lead to the accumulation of DNA damage, thus promoting cancer development (33, 34). Furthermore, failure to degrade cyclin D1 compromises the intra-S-phase checkpoint (35) and results in high expression of cyclin D1, which is often witnessed in many kinds of malignant tumors, indicating poor prognosis (36–39).

Therefore, it becomes a necessity to seek methods for degrading cyclin D1. As a potential degradation complex of cyclin D1 (32), the binding between FBX4 and cyclin D1 is still being disputed by HURI^1^, NCBI^2^, and other official websites. Furthermore, it remains unclear about the specific binding motifs of FBX4 and cyclin D1. In this review, we will focus on the FBX4–cyclin D1 binding mode from the structural biology aspect to the application aspect.

## The degradation of cyclin D1 elicits G0/G1 cell cycle arrest

Compared with cyclin D1 degradation, targeting CDK inhibition is more commonly observed clinically. The FDA has approved CDK inhibitors (CKIs) for patients with metastatic breast cancer (40). CKIs mainly act in the inhibition of RB phosphorylation and mediate cell cycle arrest. Besides, CKIs also play a role in modulating mitogenic kinase signaling and then inducing a senescence-like phenotype and enhancing cancel cell immunogenicity (41). However, CKIs usually lead to the avoidance of their conserved ATP-binding sites. Recently, researcher found that apart from CKIs that inhibiting cyclin D1, there also exist CDK degraders for degrading cyclin D1. Jiang et al. have developed Ikaros (IKZF1) and Aiolos (IKZF3) as imide-based CDK4/6 degraders in mantle cell lymphoma cell lines (42). The research and development of CDK degraders is still in the experimental stage, and patients usually are disturbed by the problems of decreased drug sensitivity in their later stages of CKI medication (43–45). Since both CDK degraders and CKIs have unavoidable defects, cyclin D1 targeted therapy is still a promising treatment. When cyclin D1 is degraded, G0/G1 arrest occurs, and then cells cease to proliferate over a period of time. The lower the amount of cyclin D1, the more likely it is to cause G0/G1 arrest.

Wu et al. (46) noted that acute cyclin D1 deficiency in the regenerating liver markedly inhibited hepatocyte proliferation after partial hepatectomy. Yang et al. (47) reported that myostatin, a transforming growth factor β-superfamily member, induces cell cycle arrest by degrading cyclin D1. Wei et al. (48) also observed that cyclin D1 degradation mediated by ERβ inhibited colon cell growth. From the opposite perspective, Li et al. (49) reported that USP5 stabilized cyclin D1 and downregulated its degradation, thus promoting glioblastoma multiforme (GBM) progression. Generally, methods for degrading cyclin D1 can be achieved through intracellular ubiquitin–proteasome degradation mechanisms that are exerted by extracellular factors.

### Extracellular factors

1. Chelating agent: Smara et al. (50) applied iron chelator deferasirox (DFX) to mantle cell lymphoma (MCL) cells, and found that DFX lead to cyclin D1 proteolysis and degradation in a mechanism that requires its phosphorylation on T286 by glycogen synthase kinase-3β (GSK3β). Several iron chelators were proved to be effective anti-cancer agents and potential therapeutic potions, leading to the ubiquitin-proteasome degradation of cyclin D1.
2. Chemical osmosis: Casanovas et al. (51) illustrated that environmental stresses induced by chemical factors can also induce cyclin D1 degradation *in vitro*. Their further research showed that osmotic stresses activate p38^SAPK2^, thereby inducing cyclin D1 degradation. Herein, chemical osmotic stress can be induced by sodium chloride (NaCl), calcium chloride (CaCl2), magnesium chloride (MgCl2), hydrogen peroxide (H2O2) and sodium arsenite (NaAsO2). ODC-antizyme, a regulator of ornithine decarboxylase (ODC), has also been demonstrated to degrade cyclin D1 by altering chemical osmosis (52).
3. Organic compounds: Several kinds of natural and synthetic organic compounds have been demonstrated to degrade cyclin D1, such as all-trans retinoic acid (RA) (53), ML364 (54), and differentiation-inducing factors (DIFs) (55). RA suppresses the G1-S transition, thereby inhibiting human bronchial epithelial cell growth. ML364 is the inhibitor of the deubiquitinase USP2, which mediates the ubiquitination of cyclin D1. DIFs induce differentiation and restrict the cell cycle in the G0/G1 phase.
4. Drugs: There is proof from several pieces of evidence that some drugs can mediate cyclin D1 degradation. Nivelle et al. (56) proved that resveratrol, epsilon viniferin, and labruscol degraded cyclin D1 in melanoma cells. Zhu et al. (57) proved that arctigenin would mediate GSK3-dependent cyclin D1 degradation in ER-positive breast cancer cells. Additionally, there are other drugs targeting cyclin D1 degradation. Luzonicoside A inhibited the proliferation and migration of cancer cells significantly more than Luzonicoside D (58). Treatment with Gambogenic acid (GNA), benzofuroxan derivative-22 (BFD-22) and Hemsleyanol D induced G0/G1 arrest (59–61). Dehydroleucodine (DhL) degraded cyclin D1 in a concentration-dependent manner (62). Simvastatin enhanced the phosphorylation and protein degradation of cyclin D1 (63, 64). Rottlerin downregulated cyclin D1 in a p21-independent mechanism (65).

### Intracellular mechanism

For the intracellular mechanism, namely, ubiquitin-proteasome degradation mechanism, two different E3 ubiquitin ligases were reported.

1. APC complex: The anaphase-promoting complex (APC) is a cell cycle regulated ubiquitin ligase. Peters et al. (66) stated in the review that cyclin D1 degradation was mediated by APC. Besides, Agami et al. reported that ionizing radiation (IR) induced cyclin D1 degradation requires an anaphase promoting complex (APC) (67).
2. AMBRA1: Activating molecule in beclin-1-regulated autophagy (AMBRA1) degrades cyclin D1 by interacting with cullin-RING family (CRL4-DDB1 complex) of E3 ubiquitin ligase, which activity depending on the phosphorylation of cyclin D1 at T286 (68, 69). Besides, CRL4^AMBRA1^ degrades three types of cyclin Ds (cyclin D1–3) through ubiquitin proteasome pathway (70).
3. SCF complex: Liu et al. (32) proposed that cyclin D1 undergoes ubiquitin-proteasome-dependent degradation *in vivo*, which is mediated by SCF complex subunit, FBX4. Besides, Okabe et al. (71) identified FBXW8 and FBXL12 as other cyclin D1 E3 ligases, which also belong to the SCF complex subunit.

Extracellular factors and intracellular ubiquitin-proteasome mechanisms mediated by APC have been thoroughly studied previously. However, the degradation of cyclin D1 specifically by the SCF complex, especially FBX4, has not been thoroughly studied. Moreover, the exact mechanism of interaction between FBX4 and cyclin D1 is still waiting to be explored. As the basis of structural biology reports on the binding of AMBRA1 and cyclin D1 is unclear yet, we would concentrate on the detailed binding mode between cyclin D1 and FBX4. Considering that CRL4^AMBRA1^ and the FBX complex have similar Cullin-RING, the analysis of the detailed binding mode between cyclin D1 and FBX4 may provide a basis for the structural biology research of AMBRA1.

## The possible combination mode between FBX4 and cyclin D1

**Figure 2:**
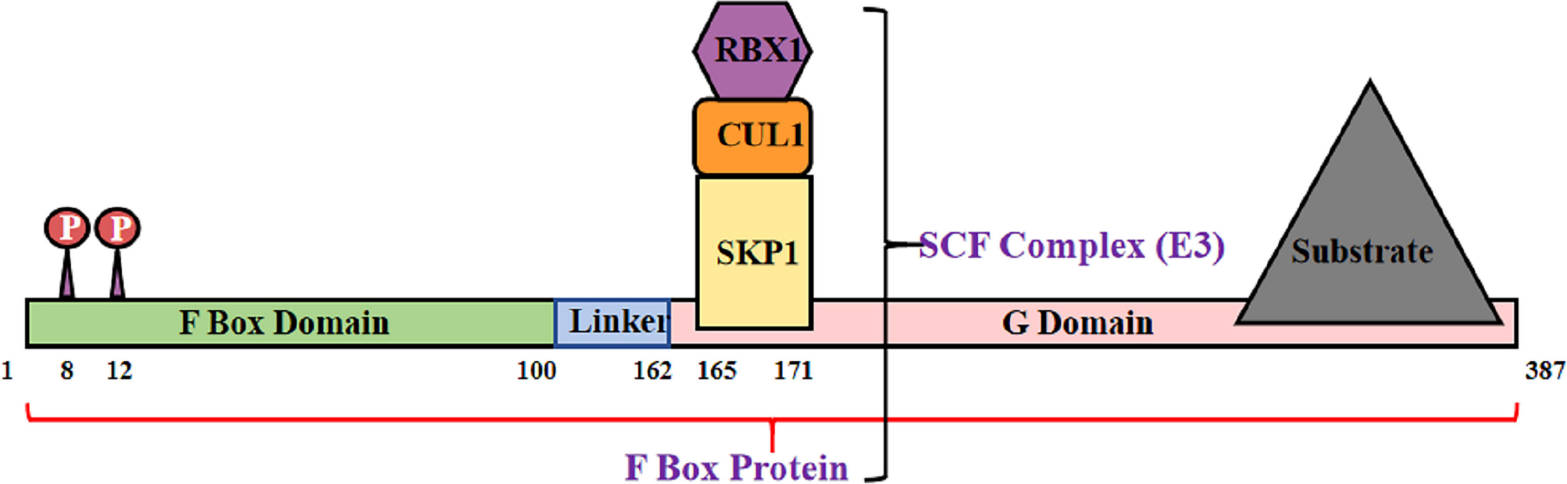
Structural diagram of FBX4. F box protein combine with Skp1, Cul1 and Rbx1, consisting SCF complex.

The Skp–Cullin–F-box (SCF) complex is an E3 ubiquitin ligase that binds to protein substrates and mediates ubiquitin-proteasome degradation. The SCF complex consists of four parts: SKP1, Cullin1 (CUL1) or cullin7 (CUL7), RBX1, or ROC1, and F box protein (72) (**Figure 2**). In this structure, the amino acid sequence specificity of the F box determines the specificity of the SCF complex. Each F-box protein contains an SKP1 binding motif as well as a specific substrate-recognition motif (73). The F-box protein family can be divided into three classes^3^: FBWs, FBLs, and FBXs. FBWs are characterized by WD-40 domains. FBLs are characterized by leucine-rich repeats and by either different protein–protein interaction modules or no recognizable motifs. FBX4, which is also named FBXO4, belongs to the FBX class.

In the following section, we would summarize the conditions required for the combination of FBX4 and cyclin D1 and propose their possible combination mode.

### With alpha B-crystalline binding and the GSK3β-mediated phosphorylation, FBX4 would bind to cyclin D1

**Figure 3:**
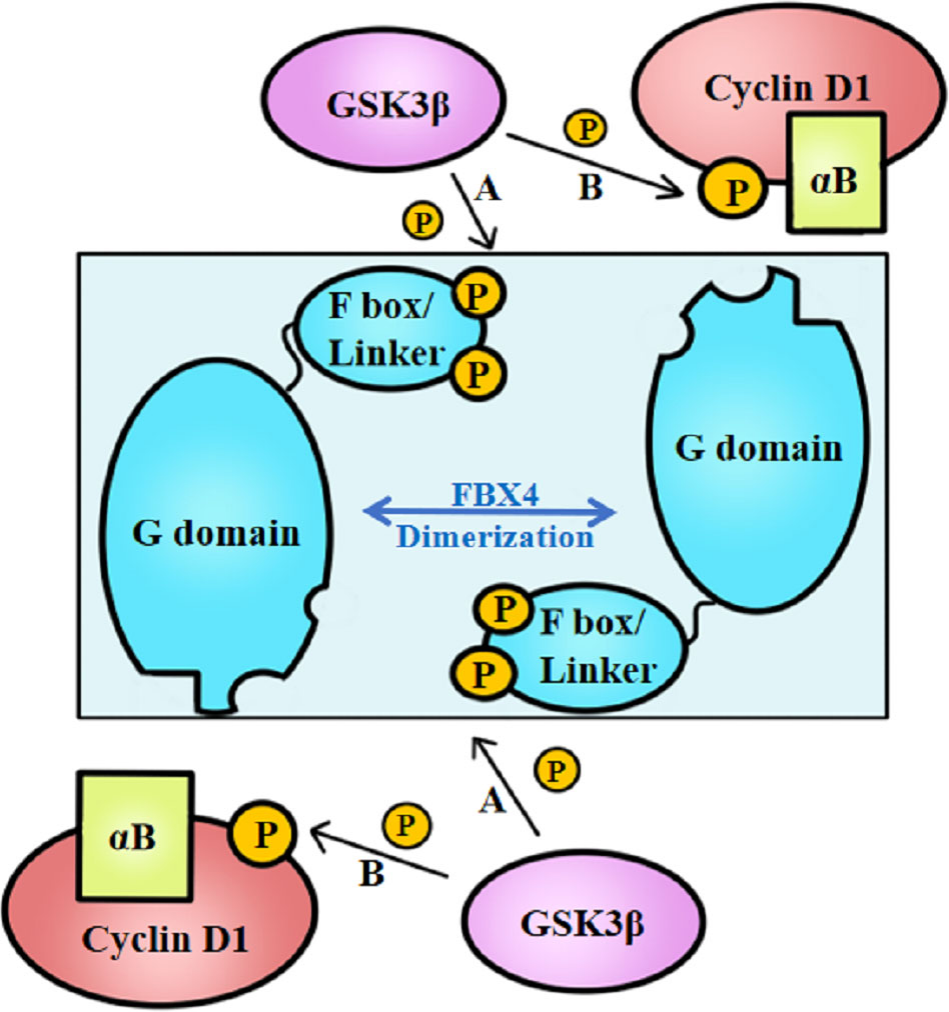
GSK3β-mediated FBX4 and cyclin D1 phosphorylation. **(A)** GSK3β phosphorylates FBX4 at Ser8 and Ser 12 of F box domain. **(B)** GSK3β phosphorylates cyclin D1 at Thr286. αB, alpha B-crystalline.

Above all, the degradation of cyclin D1 relies on FBX4 dimerization *in vivo*, which process further depends on the phosphorylation of FBX4. Barbash et al. (74) found that GSK3β mediates the phosphorylation of FBX4 at Ser8 and Ser12, thereby promoting the dimerization of FBX4 and stimulating its activity as an E3 ligase (**Figure 3A**). Compared with GSK3β inhibited cells, cells with normal GSK3β expression showed a marked decrease in growth. Barbash et al. (74) have testified that Ser8 and Ser12 phosphorylation of enhances poly-ubiquitin linked cyclin D1, while FBX4 alone or FBX4, whereas was not phosphorylated showed low degradation activities.

Besides, cyclin D1–FBX4 binding depends on alpha B-crystalline existence and cyclin D1 phosphorylation at Thr286 mediated by GSK3β (**Figure 3B**). Lin et al. (75) demonstrated that cyclin D1 binds to FBX4-alpha B-crystalline in a manner of cyclin D1 Thr286 phosphorylation *in vitro* since the cyclin D1 T286A mutant did not coprecipitate with alpha B-crystalline. What’s more, their experiments indicate that omitting alpha B-crystalline can inhibit the binding of FBX4 and cyclin D1. However, cyclin D1 precipitation mediated by FBX4 can also be detected in the absence of alpha B-crystalline, illustrating that alpha B-crystalline is not a necessity for cyclin D1–FBX4 binding.

Additionally, FBX4 associates with cyclin D1 in a relatively specific manner. Lin et al. (75) found that phosphorylated cyclin D1 peptides did not recruit other F box proteins such as FBW2 or SKP2. After IP-IB analysis of eight kinds of full-length F Box, Okabe et al. revealed that FBXW5, FBXW7, FBXW11, SKP2, and FBXL5 did not inhibit cyclin D1 expression (71).

As a supplement, the degradation of cyclin D by FBX4 may not be affected by the compensatory effect. According to the compensatory effect, if cyclin D1 alone was degraded, cyclin D2 and cyclin D3 would compensatively bind to CDK4/6 in place of cyclin D1 to promote cell cycle progression. Nevertheless, cyclin D1, cyclin D2, and cyclin D3 have similar phosphorylation sites for FBX4 binding (Thr286, Thr280, and Thr283) (3). Thus, cyclin D2 and D3 could also be degraded by FBX4 in the deduction. However, the expression of cyclin D2 and cyclin D3 is rare compared with the universal expression of cyclin D1 in most cancers (3), and that cyclin D3 plays a unique role mainly in T-cell leukemia (76). Considering the commonality of cyclin D degradation and the universality of cyclin D1, we focus on the cyclin D1–FBX4 combination.

### Conjecture of the combination mode

At present, there are few reports about the binding mechanisms between FBX4 and cyclin D1. If the binding mode of FBX4 to cyclin D1 is determined, the binding motif itself may be used as a biological component for targeted degradation of cyclin D1.

Unfortunately, the binding of FBX4 and cyclin D1 is still being doubted. From NCBI Reference Sequence NM_012176.3^4^, we found that (1): FBX4 is a protein with 387 amino acids (2); There exists two phosphorylation sites near the N-terminal of FBX4, which is coincident with the exploration of Barbash et al. (74) (3); Most importantly, the binding motif of FBX4 with another ascertained ligand—TRF1, is the peptides sequence near the C-terminal of amino acids from position 341 to 372. These information hints that the site where FBX4 binds to the substrate are supposed to be near the C-terminal.

**Figure 4:**
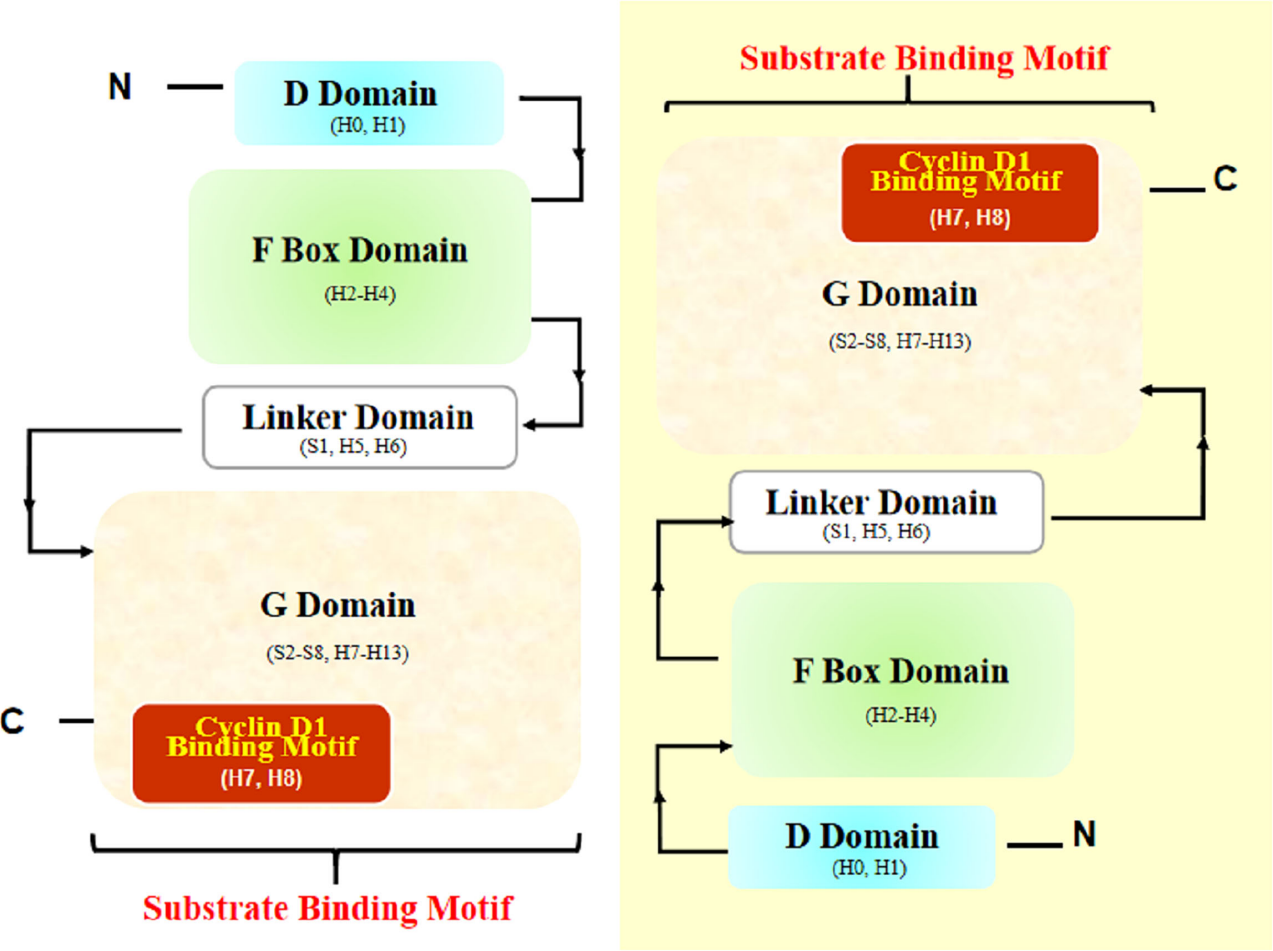
Structural diagram of FBX4 dimerization. Two FBX4 molecules dimerize in a head-to-tail manner and expose substrate binding motifs (with one FBX4 highlighted in yellow shadow). The presumed cyclin D1 binding motif is highlighted (in red shadow). H, helix; S, strand.

Another article helped a lot. In this article, Li et al. (77) provided the overall structure of the SKP1-FBX4 core complex. Structurally, FBX4 has four essential domains essential for its function in the SCF complex: N-terminal dimerization domain (D domain), F box domain, linker domain, and C-terminal substrate-binding domain (G domain) (**Figure 4**) (77). According to the structural analyses by Li et al. (77), the interaction mode between the F box domain and G domain of FBX4 is head-to-tail dimerization, which is necessary for substrate binding and ubiquitin transfer.

Thus, we can rationally deduce that the exposed end containing Helix7 (H7), Helix8 (H8), and Helix13 (H13) could be the substrate binding site. Considering the known facts that TRF1 combined with helix near the end H13 and cyclin D1 is a cyclin distinct-different from TRF1, the specifically binding motif of FBX4 and cyclin D1 could be at the other subdomain, that is, nearby H7 and H8. According to UniProt^5^, H7 and H8 are located at the positions corresponding to amino acids 193–212. However, whether the combination mode is exactly like this is waiting for experiments to testify.

**Figure 5:**
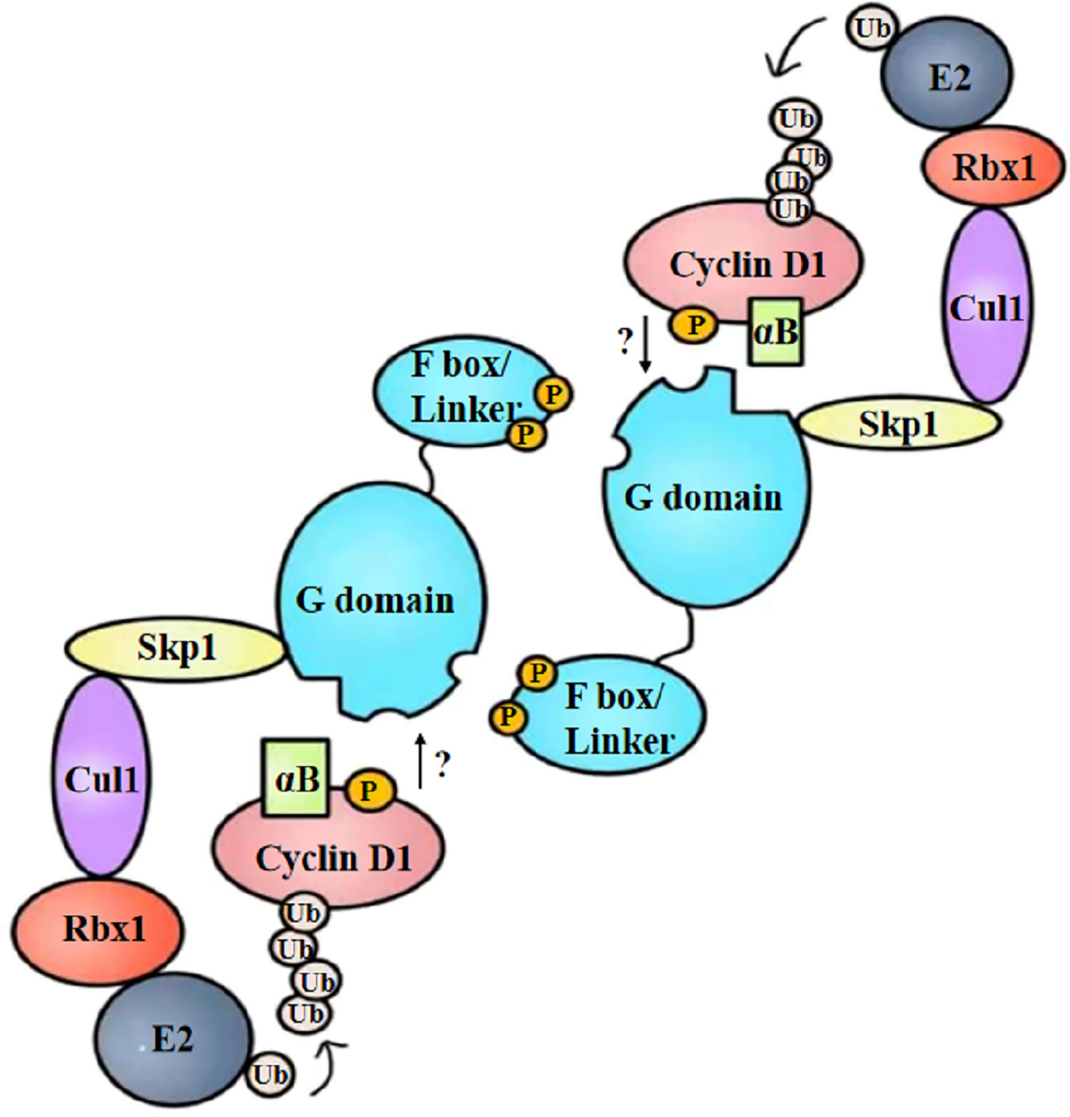
Structural diagram of FBX4-mediated cyclin D1 ubiquitination. After phosphorylation and alpha B-crystalline combination, cyclin D1 is recruited by dimerized FBX4. FBX4 also recruits the remainder of E3 ubiquitin ligase and E2 ubiquitin ligase, mediating cyclin D1 ubiquitination. αB, alpha B-crystalline; Ub, ubiquitin.

To summarize, the specific binding manner of FBX4 and cyclin D1 is supposed to be as follows: cyclin D1 is phosphorylated at Thr286 and is accompanied by alpha B-crystalline binding. Cyclin D1 phosphorylation and alpha B-crystalline are two important conditions to ensure the degradation efficiency of cyclin D1. At the same time, FBX4 proceeds head-to-tail dimerization and recruits the rest of the SCF complex such as SKP1, CUL1, and RBX1. Then phosphorylated cyclin D1 binds to FBX4 at the G domain and is eventually ubiquitinated and degraded by the proteasome (**Figure 5**). As the specific binding motif between cyclin D1 and FBX4 has not been certified, the arrow from cyclin D1 to the G domain remains a question mark.

## Application of FBX4–cyclin D1 combination in photoswitching PROTAC

As for the application of ubiquitin mediated protein degradation, Luo et al. (78) reviewed major advances in PROteolysis TArgeting Chimeras (PROTAC), which mediates the transfer of poly-ubiquitin (Ub) chains from E3 ligase to the protein of interest (POI). At present, two powerful PROTAC candidates have entered phase 1/2 clinical trials developed by the Arvinas company, namely, ARV-110 targeting androgen receptors (AR) for treating prostate cancer (79), and ARV-471 targeting estrogen receptors (ER) for treating breast cancer (80). Although the traditional PROTAC has presented a great application value, there is still the problem of poor controllability and off-target effect. If the reversible optical switch can be added, these problems will be greatly solved. Among different controllable PROTACs, the photoswiching PROTAC strategy is reversible. By switching the light wavelength irradiating the controlling element of PROTAC, PROTAC can bind with POI and then mediate ubiquitin proteasome degradation. Otherwise, PROTAC would be separated from POI and then POI degradation would stop (81). The switch of a specific wavelength depends on the characteristics of the optical control element.

Photodynamic therapy is becoming a new hotspot in recent years. Ye et al. (82) constructed an optogenetic switch to regulate Fc-adiponectin expression in photosensitive cells by using far-red light and effectively reduced blood lipid and blood sugar, thus alleviating the symptoms of insulin resistance. Later, the team developed the REDMAP system (83), a red/far-red optogenetic switch used to regulate insulin expression and hypoglycemia in diabetic mice and rats. Their academic achievements indicate that the light-controlled system has a great application prospect. The NUDT IGEM team^6^ has made an achievement in integrating Trim21, a cytosolic ubiquitin ligase and antibody receptor with a blue light controlled model (CRY2 and its ligand CIB1) to target GFP degradation.

**Figure 6:**
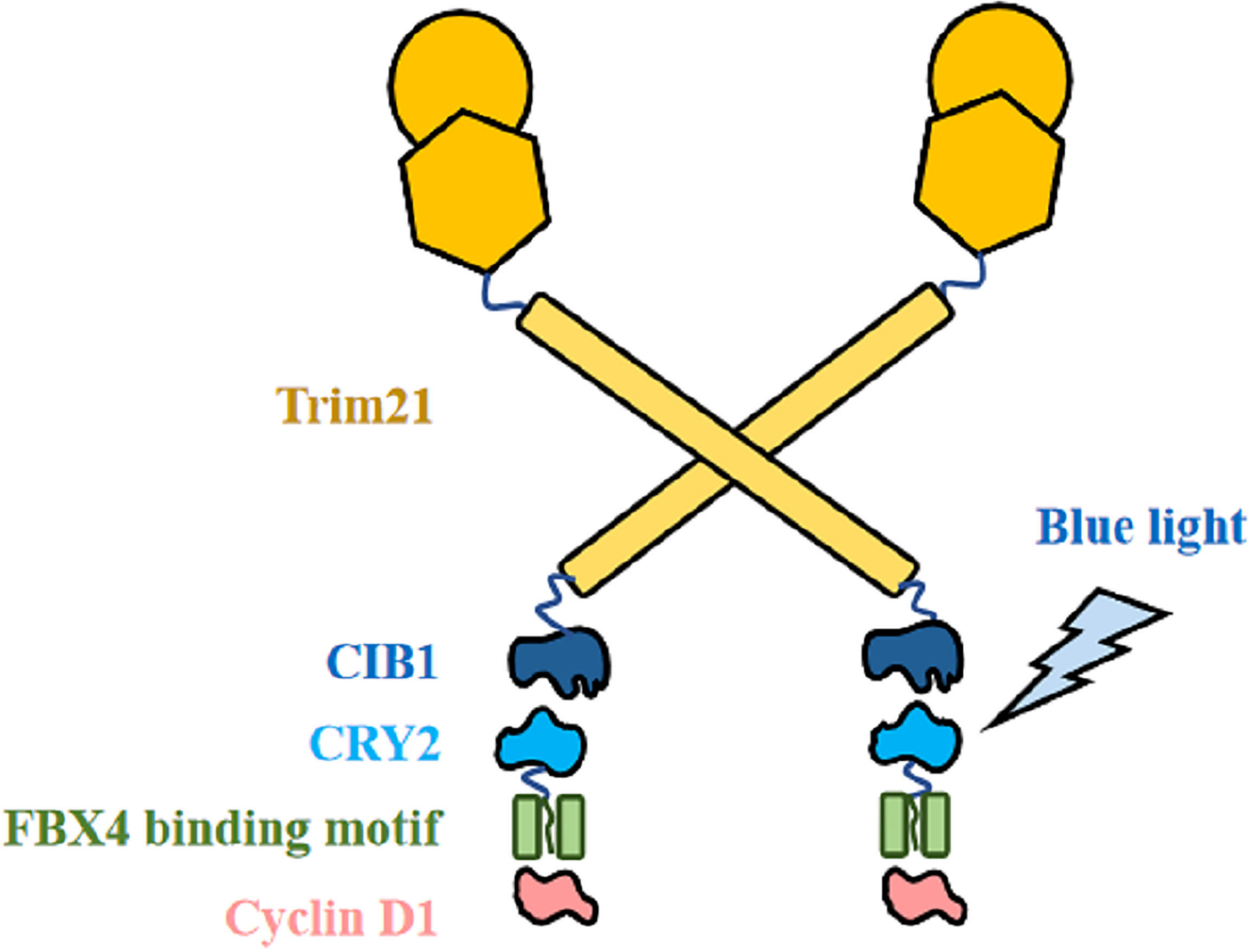
CIB1–FBX4 system. FBX4 binding motif captures cyclin D1. After blue light stimulation, CIB1 combines with CRY2 (84), leading to Trim21-mediated cyclin D1 degradation.

Based on the theoretical basis above, we then propose to modify it into a cyclin D1 degradation model by proposing the CIB1-FBX4 system. In this system, blue light mediates the combination of two photosensitive proteins of Arabidopsis, CIB1 and CRY2, by causing them to deform (84). As shown in **Figure 6**, CIB1 and Trim21 are designed by linking together, whereas CRY2 and the capture sequence of FBX4 are linked together with a segment of flexible peptide. If there were no light signal, the FBX4 binding motif could capture target cyclin D1 but does not cause its degradation. Upon blue light stimulation, CIB1 can bind to CRY2, which is then accompanied by the binding of Trim21 to cyclin D1, and subsequently the degradation of cyclin D1 (**Figure 6**). If the exact binding motif of FBX4 was integrated into their optical control model, rapid and efficient blue light-controlled cyclin D1 degradation would be achieved. Thus, we can synchronize the cycle of the tumor cells at the G0 phase. On the condition that the cycle of the tumor cells is synchronized before M phase, they stop proliferation and diffusion. This strategy is especially suitable for superficial cancers such as esophageal squamous carcinoma, because these lesions are easily irradiated by light. For the specific application, just take esophageal squamous carcinoma as an example. Specific implementation methods include modifying gastroscopy apparatus and transfecting the CIB1–FBX4 system into cancer cells. The proliferation of cancer cells will be slowed down by light therapy, which acts in a similar manner to gastroscopy.

The outstanding features of the CIB1–FBX4 system include more reversible and sufficient degradation than the FBX4 whole sequence. It is inevitable that there are normal cells transfected with the CIB1–FBX4 system that experience G0/G1 arrest temporarily. However, the expression of cyclin D1 will be restored after light removal, and the normal cells will resume normal division after a while. To the greatest extent, it avoids “accidental injury.” In addition, it solves problems such as insufficient regulation ability of existing protein-targeted degradation tools on intracellular coding proteins, influence on normal physiological functions of cells, and limited application *in vivo* environment.

It is worth noting that there are drawbacks to this approach. As CIB1 and CRY2 come from *Arabidopsis thaliana*, a plant, it is unclear whether the system will cause immunological rejection *in vivo*. Therefore, the system is expected to be tested in organoids with an immune microenvironment. More light-controlled components are expected to be discovered and applied as well.

## Author contributions

SC completed all gathering and arranging work under the guidance of LL. All authors contributed to the article and approved the submitted version.

## Conflict of interest

The authors declare that the research was conducted in the absence of any commercial or financial relationships that could be construed as a potential conflict of interest.

## Publisher’s note

All claims expressed in this article are solely those of the authors and do not necessarily represent those of their affiliated organizations, or those of the publisher, the editors and the reviewers. Any product that may be evaluated in this article, or claim that may be made by its manufacturer, is not guaranteed or endorsed by the publisher.

## Footnotes

1
http://www.interactome-atlas.org/search#

2
https://www.ncbi.nlm.nih.gov/Structure/cdd/cddsrv.cgi?uid=212555

3
https://www.genecards.org/cgi-bin/carddisp.pl?gene=FBXO4

4
https://www.ncbi.nlm.nih.gov/nuccore/NM_012176.3

5
https://www.uniprot.org/uniprot/Q9UKT5/protvista

6
https://2020.igem.org/Team:NUDT_CHINA/Design
